# Supplementary material for: TREX reveals proteins that bind to specific RNA regions in living cells
Source: Nat Methods. 2024 Feb 19;21(3):423–34. doi: 10.1038/s41592-024-02181-1 (PMC10927567; doi:10.1038/s41592-024-02181-1)
Supplement: Supplementary file 1 — Supplementary Tables and associated descriptions/legends. [file 41592_2024_2181_MOESM1_ESM.pdf]

nature methods

Article https://doi.org/10.1038/s41592-024-02181-1

TREX reveals proteins that bind to specific RNA regions in living cells

In the format provided by the authors and unedited

Supplementary Information

Supplementary material for this manuscript consists of supplementary tables 1-3, and a supplementary excel file containing supplementary datasets (Datasets 1-11).

Supplementary Tables:

Supplementary Table 1: List of TREX antisense DNA oligonucleotides used in this study for RNase H-mediated depletion of indicated target RNAs.

**TREX tiling antisense oligo**	**Sequence**
U1	
U1_Trex_1	CAGGGGAAAGCGCGAACGCAGTCCCCCACTACCACAAATTATGCAGTCGAGTTTCCCACAT
U1_Trex_2	TTTGGGGAAATCGCAGGGGTCAGCACATCCGGAGTGCAATGGATAAGCCTCGCCCTGGGAA
U1_Trex_3	AAACCACCTTCGTGATCATGGTATCTCCCCTGCCAGGTAAGTAT
NORAD	
NORAD-ND4_Trex_1	TCCTCCTTCCCCCTGAACCAGCACAAATATCAAAATGGGTACTGCTCAGAGAATTGATCA
NORAD-ND4_Trex_2	ATTTCCAAGACAATATTTCATATCAAAAGGATAGCTACAAAATGTGTTATTTACTACCTC
NORAD-ND4_Trex_3	GACTTAAGTTTGTCCGCTATATACACAGTAGGGTTGAATAAACTACACACATGTAACAAT
NORAD-ND4_Trex_4	GGTTATATCTGATAGTGTCTTCTAAATACGAACATTCTGGTCTAGAACCCTTCATTCCTT
NORAD-ND4_Trex_5	CCAACTCCTCTCCACCACCAACCTGATGGATATAGGCACGTGTCACTTAGAGCTGATGTT
NORAD-ND4_Trex_6	ATCTCACTTCCAAAAGTCCTTTTCAGAAGACAGCCTTTCTACAAATTTTAACAAAGTGTA
NORAD-ND4_Trex_7	AGAGACTAGATATTATAAAATCGGGACTAATTTGTCCGTTATATATACAATATATACAGT
NORAD-ND4_Trex_8	ATAGCAAAGTTAAATGAAATGCATGTAACATATACACAATGGATTAAGCTGAAATTTTCT
NORAD-ND4_Trex_9	CAATTCCACTTCCAAACAGCATTTCCCATCAGTTTTTAAAAGC
45S	
45S_Trex_1	CGCGAGGGCCGGGACCCCGGGTGGCCGCCCCACCGGGGCCCGCGCGGCCAACCCCCGGGA
45S_Trex_2	CGGGGACCGGCGGGCCACGGGCCCGGCTCGGCGCGGCCGCCTCCGCGGCTCCCAAACCAC
45S_Trex_3	GCTCCCCGGACCCCGTCCCGGCCCGGAGCGGACGAGCCGCCCCGGCGGTGAACGGGGAGG
45S_Trex_4	AGGCGGGAACCGAAGAAGCGGGGCGCGCCGACCGGGGTCGCGCGCCCTCCCCCCCACCCC

45S_Trex_5	CACCACCACGCCCGCGGTCGGCGGGAGAGGCCGGGAGGGAGGAAGACGAACGGAAGGACG
45S_Trex_6	GACGGCGCCGGACGCGCACGCCCCGCCGGGCCCCCGCACGCACGCGCGCGCGCGCGC
45S_Trex_7	GGACAAACCCTTGTGTCGAGGGCTGACTTTCAATAGATCGCAGCGAGGGAGCTGCTCTGC
45S_Trex_8	TACGTACGAAACCCCGACCCAGAAGCAGGTCGTCTACGAATGGTTTAGCGCCAGGTTCCC
45S_Trex_9	CACGAACGTGCGGTGCGTGACGGGCGAGGGGGCGGCCGCCTTTCCAGCCGCGCCCCGTTT
45S_Trex_10	CCCAGGACGAAGGGCACTCCGCACCGGACCCCGGTCCCGGCGCGCGGCGGGGCACGCGCC
45S_Trex_11	CTCCCGCGCGCGCGGGGCGCGTGGAGGGGGGGCGGCCCGCCGGCGGGGACAGGCGGGGG
45S_Trex_12	ACCGGCTATCCGAGGCCAACCGAGGCTCCGCGGCGCTGCCGTATCGTTCCGCCTGGGCGG
45S_Trex_13	GATTCTGACTTAGAGGCGTTCAGTCATAATCCCACAGATGGTAGCTTCGCCCCATTGGCT
45S_Trex_14	CCTCAGCCAAGCACATACACCAAATGTCTGAACCTGCGGTTCCTCTCGTACTGAGCAGGA
45S_Trex_15	TTACCATGGCAACAACACATCATCAGTAGGGTAAAACTAACCTGTCTCACGACGGTCTAA
45S_Trex_16	ACCCAGCTCACGTTCCCTATTAGTGGGTGAACAATCCAACGCTTGGTGAATTCTGCTTCA
45S_Trex_17	CAATGATAGGAAGAGCCGACATCGAAGGATCAAAAAGCGACGTCGCTATGAACGCTTGGC
45S_Trex_18	CGCCACAAGCCAGTTATCCCTGTGGTAACTTTTCTGACACCTCCTGCTTAAAACCCAAAA
45S_Trex_19	GGTCAGAAGGATCGTGAGGCCCCGCTTTCACGGTCTGTATTCGTACTGAAAATCAAGATC
45S_Trex_20	AAGCGAGCTTTTGCCCTTCTGCTCCACGGGAGGTTTCTGTCCTCCCTGAGCTCGCCTTAG
45S_Trex_21	GACACCTGCGTTACCGTTTGACAGGTGTACCGCCCCAGTCAAACTCCCCACCTGGCACTG
45S_Trex_22	TCCCCGGAGCGGGTCGCGCCCGGCCGGCGCGCGGCCGGGCGCTTGGCGCCAGAAGCGAGA
45S_Trex_23	GCCCCTCGGGGCTCGCCCCCCCGCCTCACCGGGTCAGTGAAAAAACGATCAGAGTAGTGG
45S_Trex_24	TATTTCACCGGCGGCCCGCAGGGCCGGCGGACCCCGCCCCGGGCCCCTCGCGGGGACACC
45S_Trex_25	GGGGGGGCGCCGGGGGCCTCCCACTTATTCTACACCTCTCATGTCTCTTCACCGTGCCAG
45S_Trex_26	ACTAGAGTCAAGCTCAACAGGGTCTTCTTTCCCCGCTGATTCCGCCAAGCCCGTTCCCTT
45S_Trex_27	GGCTGTGGTTTCGCTGGATAGTAGGTAGGGACAGTGGGAATCTCGTTCATCCATTCATGC
45S_Trex_28	GCGTCACTAATTAGATGACGAGGCATTTGGCTACCTTAAGAGAGTCATAGTTACTCCCGC
45S_Trex_29	CGTTTACCCGCGCTTCATTGAATTTCTTCACTTTGACATTCAGAGCACTGGGCAGAAATC
45S_Trex_30	ACATCGCGTCAACACCCGCCGCGGGCCTTCGCGATGCTTTGTTTTAATTAAACAGTCGGA
45S_Trex_31	TTCCCCTGGTCCGCACCAGTTCTAAGTCGGCTGCTAGGCGCCGGCCGAGGCGAGGCGCCG
45S_Trex_32	CGCGGAACCGCGGCCCCGGGGGCGGACCCGGCGGGGGGGACCGGCCCGCGGCCCCTCCGC

45S_Trex_33	CGCCCGCCGCCGCCGCCGCGCGCCGAGGAGGAGGGGGGAACGGGGGGCGGACGGGGCCGG
45S_Trex_34	GGGGGTAGGGCGGGGGGACGAACCGCCCCGCCCCGCCGCCCGCCGACCGCCGCCGCCCGA
45S_Trex_35	CCGCTCCCCGCCCCCAGCGGACGCGCGCGCGACGAGACGTGGGGTGGGGGGGGGGCGCG
45S_Trex_36	CCGGCGCCCGCCGGGCTCCCCGGGGGCGGCCGCGACGCCCGCCGCAGCTGGGGCGATCCA
45S_Trex_37	CGGGAAGGGCCCGGCTCGCGTCCAGAGTCGCCGCCGCCGCCGGCCCCCCGGGTGCCCGGG
45S_Trex_38	CCCCCCTCGCGGGGGACCGTGCCCCCGCCGCCGGGGCCCCGCGGCGGGCCGCCGCCGGCC
45S_Trex_39	CCTGCCGCCCCGACCCTTCTCCCCCCGCCGCCGCCCCCACGCGGCGCTCCCCCGGGGAGG
45S_Trex_40	GGGGAGGACGGGGAGCGGGGGAGAGAGAGAGAGAGAGGGCGCGGGGCGGGGAGGGAGCGA
45S_Trex_41	GCGGCGCGCGCGGGGTGGGGCGGGGGAGGGCCGCGAGGGGGGTGCCCCGGGCGTGGGGGG
45S_Trex_42	GGCGGCGGCGCCTCGTCCAGCCGCGGCGCGCGCCCAGCCCCGCTTCGCGCCCCAGCCCGA
45S_Trex_43	CCGACCCAGCCCTTAGAGCCAATCCTTATCCCGAAGTTACGGATCCGGCTTGCCGACTTC
45S_Trex_44	CCTTACCTACATTGTTCCAACATGCCAGAGGCTGTTCACCTTGGAGACCTGCTGCGGATA
45S_Trex_45	TGGGTACGGCCCGGCGCGAGATTTACACCCTCTCCCCCGGATTTTCAAGGGCCAGCGAGA
45S_Trex_46	GCTCACCGGACGCCGCCGGAACCGCGACGCTTTCCAAGGCACGGGCCCCTCTCTCGGGGC
45S_Trex_47	GAACCCATTCCAGGGCGCCCTGCCCTTCACAAAGAAAAGAGAACTCTCCCCGGGGCTCCC
45S_Trex_48	GCCGGCTTCTCCGGGATCGGTCGCGTTACCGCACTGGACGCCTCGCGGCGCCCATCTCCG
45S_Trex_49	CCACTCCGGATTCGGGGATCTGAACCCGACTCCCTTTCGATCGGCCGAGGGCAACGGAGG
45S_Trex_50	CCATCGCCCGTCCCTTCGGAACGGCGCTCGCCCATCTCTCAGGACCGACTGACCCATGTT
45S_Trex_51	CAACTGCTGTTCACATGGAACCCTTCTCCACTTCGGCCTTCAAAGTTCTCGTTTGAATAT
45S_Trex_52	TTGCTACTACCACCAAGATCTGCACCTGCGGCGGCTCCACCCGGGCCCGCGCCCTAGGCT
45S_Trex_53	TCAAGGCTCACCGCAGCGGCCCTCCTACTCGTCGCGGCGTAGCGTCCGCGGGGCTCCGGG
45S_Trex_54	GGCGGGGAGCGGGGCGTGGGCGGGAGGAGGGGAGGAGGCGTGGGGGGGGGGCGGGGGAA
45S_Trex_55	GGACCCCACACCCCCGCCGCCGCCGCCGCCGCCGCCCTCCGACGCACACCACACGCGCGC
45S_Trex_56	GCGCGCGCGCCGCCCCCGCCGCTCCCGTCCACTCTCGACTGCCGGCGACGGCCGGGTATG
45S_Trex_57	GGCCCGACGCTCCAGCGCCATCCATTTTCAGGGCTAGTTGATTCGGCAGGTGAGTTGTTA
45S_Trex_58	CACACTCCTTAGCGGATTCCGACTTCCATGGCCACCGTCCTGCTGTCTATATCAACCAAC
45S_Trex_59	ACCTTTTCTGGGGTCTGATGAGCGTCGGCATCGGGCGCCTTAACCCGGCGTTCGGTTCAT
45S_Trex_60	CCCGCAGCGCCAGTTCTGCTTACCAAAAGTGGCCCACTAGGCACTCGCATTCCACGCCCG

45S_Trex_61	GCTCCACGCCAGCGAGCCGGGCTTCTTACCCATTTAAAGTTTGAGAATAGGTTGAGATCG
45S_Trex_62	TTTCGGCCCCAAGACCTCTAATCATTCGCTTTACCGGATAAAACTGCGTGGCGGGGGTGC
45S_Trex_63	GTCGGGTCTGCGAGAGCGCCAGCTATCCTGAGGGAAACTTCGGAGGGAACCAGCTACTAG
45S_Trex_64	ATGGTTCGATTAGTCTTTCGCCCCTATACCCAGGTCGGACGACCGATTTGCACGTCAGGA
45S_Trex_65	CCGCTACGGACCTCCACCAGAGTTTCCTCTGGCTTCGCCCTGCCCAGGCATAGTTCACCA
45S_Trex_66	TCTTTCGGGTCCTAACACGTGCGCTCGTGCTCCACCTCCCCGGCGCGGCGGGCGAGACGG
45S_Trex_67	GCCGGTGGTGCGCCCTCGGCGGACTGGAGAGGCCTCGGGATCCCACCTCGGCCGGCGAGC
45S_Trex_68	GCGCCGGCCTTCACCTTCATTGCGCCACGGCGGCTTTCGTGCGAGCCCCCGACTCGCGCA
45S_Trex_69	CGTGTTAGACTCCTTGGTCCGTGTTTCAAGACGGGTCGGGTGGGTAGCCGACGTCGCCGC
45S_Trex_70	CGACCCCGTGCGCTCGCTCCGCCGTCCCCCTCTTCGGGGGACGCGCGCGTGGCCCCGAGA
45S_Trex_71	GAACCTCCCCCGGGCCCGACGGCGCGACCCGCCCGGGGCGCACTGGGGACAGTCCGCCCC
45S_Trex_72	GCCCCCCGACCCGCGCGCGGCACCCCCCCCGTCGCCGGGGCGGGGGCGCGGGGAGGAGGG
45S_Trex_73	GTGGGAGAGCGGTCGCGCCGTGGGAGGGGTGGCCCGGCCCCCCCACGAGGAGACGCCGGC
45S_Trex_74	GCGCCCCCGCGGGGGAGACCCCCCTCGCGGGGGATTCCCCGCGGGGGTGGGCGCCGGGAG
45S_Trex_75	GGGGGAGAGCGCGGCGACGGGTCTCGCTCCCTCGGCCCCGGGATTCGGCGAGTGCTGCTG
45S_Trex_76	CCGGGGGGGCTGTAACACTCGGGGGGGGTTTCGGTCCCGCCGCCGCCGCCGCCGCCGCCA
45S_Trex_77	CCGCCGCCGCCGCCGCCGCCCCGACCCGCGCGCCCTCCCGAGGGAGGACGCGGGGCCGGG
45S_Trex_78	GGGCGGAGACGGGGGAGGAGGAGGACGGACGGACGGACGGACGGGGCCCCCCGAGCCACC
45S_Trex_79	TTCCCCGCCGGGCCTTCCCAGCCGTCCCGGAGCCGGTCGCGGCGCACCGCCGCGGTGGAA
45S_Trex_80	ATGCGCCCGGCGGCGGCCGGTCGCCGGTCGGGGGACGGTCCCCCGCCGACCCCACCCCCG
45S_Trex_81	GCCCCGCCCGCCCACCCCCGCACCCGCCGGAGCCCGCCCCCTCCGGGGAGGAGGAGGAGG
45S_Trex_82	GGCGGCGGGGGAAGGGAGGGCGGGTGGAGGGGTCGGGAGGAACGGGGGGCGGGAAAGATC
45S_Trex_83	CGCCGGGCCGCCGACACGGCCGGACCCGCCGCCGGGTTGAATCCTCCGGGCGGACTGCGC
45S_Trex_84	GGACCCCACCCGTTTACCTCTTAACGGTTTCACGCCCTCTTGAACTCTCTCTTCAAAGTT
45S_Trex_85	CTTTTCAACTTTCCCTTACGGTACTTGTTGACTATCGGTCTCGTGCCGGTATTTAGCCTT
45S_Trex_86	AGATGGAGTTTACCACCCGCTTTGGGCTGCATTCCCAAGCAACCCGACTCCGGGAAGACC
45S_Trex_87	CGGGCCCGGCGCGCCGGGGGCCGCTACCGGCCTCACACCGTCCACGGGCTGGGCCTCGAT
45S_Trex_88	CAGAAGGACTTGGGCCCCCCACGAGCGGCGCCGGGGAGCGGGTCTTCCGTACGCCACATG

45S_Trex_89	TCCCGCGCCCCGCCGCGGGGCGGGGATTCGGCGCTGGGCTCTTCCCTGTTCACTCGCCGT
45S_Trex_90	TACTGAGGGAATCCTGGTTAGTTTCTTCTCCTCCGCTGACTAATATGCTTAAATTCAGCG
45S_Trex_91	GGTCGCCACGTCTGATCTGAGGTCGCGTCTCGGAGGGGGACGGGCCGCTCGGCGGACGGA
45S_Trex_92	CGGACGGAATCGCGCCGGCCCGACCGCCCGCCCGACGCTCCGTCGGGAGACGGGCCCGGC
45S_Trex_93	GAGGGGGAGAGGCGACGGGAGAGAGAGCGCGCGGCCGGCGGCACCCCCGCGCCGCCCCGC
45S_Trex_94	CGGAGCGGGACGACCGGAGGGAGGGGCACGGGCCGGGGGCGGGACGGGCGCCGCACGCCC
45S_Trex_95	CGACCCGTCTCCCCCGCGGAGGTCGGGGGGACGGGTCCGAGGACGCGGCGGCGGAGCCGC
45S_Trex_96	CCCGCCCCGACGCGGAAGCTCGGGACGGGGCCCCGGCGCGGCGCGGCGCGGCCGCGAGCC
45S_Trex_97	GGAGGCGGGCGCGCGACGGCGGACGACACCGCGGCGTCCCGCGGGTCGCCGCCGGGGACA
45S_Trex_98	CGCGAACCCCGGCGCCGCGGCCACGGGCGCGGCCGGGCGGGCCGCGGGGCGGGCTCCCGG
45S_Trex_99	CCCCGGCCGACGCGCCGCGAGGCGAGCCGGGCGGGCGGGCGCGCGTACGCGCGGGGAGGG
45S_Trex_100	CGAGGAGGACGGGCGGGGCCTCGGAGGAGGGGCGGCGGGGAGGAGGAGGGGCGCGGGAGC
45S_Trex_101	GGCGGTCGGCCGGACGCCGGGCCGCCACCGGGGGCGGGCGGCGAACCGCGGCGACCGGGA
45S_Trex_102	CGCGCTCCCCCGACCCTCTCTCCCCGCCGGCACCCTTCCCCTTCCGGACCCGCCTTCCTC
45S_Trex_103	CTCCCCCACCACCACACCGCACGCAACACGCCCCCACCGCCGACGACGCGCGACGACGAC
45S_Trex_104	GACGACGACGGGCACGGGACCTTCCACCCGGCCGGGGCCGACGAACCCCGAACCCCGAGC
45S_Trex_105	CGCGCGCGGCGCGAGGGAGCCCCCCGAGGGAGGAACCCGGACCGCAGGCGGCGGCCACGG
45S_Trex_106	GAACTCGGCCCGAGCCGGCTCTCTCTTTCCCTCTCCGTCTTCGCGGGCGGCGGCGGCGCC
45S_Trex_107	GCCCTCCCCGTCTCTCTCAGCCGGGCGCGCCCCCCTCTCCCCCCCGCCACCCGACGCGTG
45S_Trex_108	ACCACGCAGGGCCCGCGGGGGGAGGGGGAAGGGGCGGGCGCGGCGGCAAGAGGAGGGCGG
45S_Trex_109	ACGCCGCCGGGTCTGCGCTTAGGGGGACGGAGGGCCCCCGGCGGGCCCTGCGAGGGAACC
45S_Trex_110	CCCAGCCGCGCACCCCGAGGAGCCCGGAGGCACCCCCGGGGGCGATTGATCGGCAAGCGA
45S_Trex_111	CGCTCAGACAGGCGTAGCCCCGGGAGGAACCCGGGGCCGCAAGTGCGTTCGAAGTGTCGA
45S_Trex_112	TGATCAATGTGTCCTGCAATTCACATTAATTCTCGCAGCTAGCTGCGTTCTTCATCGACG
45S_Trex_113	CACGAGCCGAGTGATCCACCGCTAAGAGTCGTACGAGGTCGATTTGGCGAGGGCGCTCCC
45S_Trex_114	GACGACGCACCGGGAGGAGGCCCTTCCTGGCGCGGCACGTCCCCCCCCCCCCGCCCAAG
45S_Trex_115	AGGAGAGGGGGTTGCCTCAGGCCGGCCAGACGAGACAGCAAACGGGACCGGACTCCGGAG
45S_Trex_116	AGGGGTCGGAAGGTTTCACACCACGGGGAGGCGCGCGCCGCCCACGCGGGGGCGAGCGCG

45S_Trex_117	GACACCACCCCACAGGCGCCCGGGGGTTCCCGCCCCCACGGCGCGGGGCGCACGCCACAC
45S_Trex_118	GCGCGGCAGGCGCGCGACGGCCGCCGGGTAAAGCCCCCACCCGACGGCCGCCGCGGCGGC
45S_Trex_119	GGCGGCGGCGCGGCCCCGGCCGGGGAGCGGAGTCCGCGGTGGAGGCGCGGGAGGGGCCGG
45S_Trex_120	GCCCCTCCCGACGGGACTCCCCCGCGGGCCCACCACCGCCCCCGACCCACGGGCGGACGG
45S_Trex_121	GCGATCCCCCCAAGGGGTCTTTAAACCTCCGCGCCGGAACGCGCTAGGTACCTGGACGGC
45S_Trex_122	GGGGGGGCGGACGAGGAGGCGGGGGAGGGGACCGGCGTCCGGCCCCCGACCCTCGAGACG
45S_Trex_123	CCCTAGCGGGAAGGCCGGGGAGAGCGAGCGGGGCCGTGCCCGGCGGCGCGGAGCGGCGCG
45S_Trex_124	GCGGAGGCGACGGGAATCCGGCCGGCCCCGAAGACGGGGAGCCGGCGCGGCGGGGCCGGA
45S_Trex_125	CGACGGGCCCCGGCGGGGAGGAGGGCACCGAGACCCCCCCAGACCCGCCGCGACGCCGCC
45S_Trex_126	GAGAACCGCCCCCGCGCCCGCCGACACCCACGTCGTCGGGGCCGCGGCCGGGGACCGCTC
45S_Trex_127	CCCGCCGCCCGCCGGCCCCACGACACGCGCACACCAACGACACGCCCTTCTTTCTCTTTC
45S_Trex_128	TCTCTCTCTCTCTCTCTCCCCCGTCTCCCTCCCGAGTTCTCCGGCTCTCGCGGCCGGCGG
45S_Trex_129	GGCCGGGCGGCGAACGAACGAGCGAGCGAACGAACGGGCACGCGGGCCCCGCCCGCGCAC
45S_Trex_130	GCGCCGCGTCGCGGTGGGGGGGTGGGTGTGCGGAGGGAAGCGCGCGGCGGCGGCGGCGCC
45S_Trex_131	GCCGCGGGCCTCGCCCTCCGGGCTCCGTTAATGATCCTTCCGCAGGTTCACCTACGGAAA
45S_Trex_132	CCTTGTTACGACTTTTACTTCCTCTAGATAGTCAAGTTCGACCGTCTTCTCAGCGCTCCG
45S_Trex_133	CCAGGGCCGTGGGCCGACCCCGGCGGGGCCGATCCGAGGGCCTCACTAAACCATCCAATC
45S_Trex_134	GGTAGTAGCGACGGGCGGTGTGTACAAAGGGCAGGGACTTAATCAACGCAAGCTTATGAC
45S_Trex_135	CCGCACTTACTGGGAATTCCTCGTTCATGGGGAATAATTGCAATCCCCGATCCCCATCAC
45S_Trex_136	GAATGGGGTTCAACGGGTTACCCGCGCCTGCCGGCGTAGGGTAGGCACACGCTGAGCCAG
45S_Trex_137	TCAGTGTAGCGCGCGTGCAGCCCCGGACATCTAAGGGCATCACAGACCTGTTATTGCTCA
45S_Trex_138	ATCTCGGGTGGCTGAACGCCACTTGTCCCTCTAAGAAGTTGGGGGACGCCGACCGCTCGG
45S_Trex_139	GGGTCGCGTAACTAGTTAGCATGCCAGAGTCTCGTTCGTTATCGGAATTAACCAGACAAA
45S_Trex_140	TCGCTCCACCAACTAAGAACGGCCATGCACCACCACCCACGGAATCGAGAAAGAGCTATC
45S_Trex_141	AATCTGTCAATCCTGTCCGTGTCCGGGCCGGGTGAGGTTTCCCGTGTTGAGTCAAATTAA
45S_Trex_142	GCCGCAGGCTCCACTCCTGGTGGTGCCCTTCCGTCAATTCCTTTAAGTTTCAGCTTTGCA
45S_Trex_143	ACCATACTCCCCCCGGAACCCAAAGACTTTGGTTTCCCGGAAGCTGCCCGGCGGGTCATG
45S_Trex_144	GGAATAACGCCGCCGCATCGCCGGTCGGCATCGTTTATGGTCGGAACTACGACGGTATCT

45S_Trex_145	GATCGTCTTCGAACCTCCGACTTTCGTTCTTGATTAATGAAAACATTCTTGGCAAATGCT
45S_Trex_146	TTCGCTCTGGTCCGTCTTGCGCCGGTCCAAGAATTTCACCTCTAGCGGCGCAATACGAAT
45S_Trex_147	GCCCCCGGCCGTCCCTCTTAATCATGGCCTCAGTTCCGAAAACCAACAAAATAGAACCGC
45S_Trex_148	GGTCCTATTCCATTATTCCTAGCTGCGGTATCCAGGCGGCTCGGGCCTGCTTTGAACACT
45S_Trex_149	CTAATTTTTTCAAAGTAAACGCTTCGGGCCCCGCGGGACACTCAGCTAAGAGCATCGAGG
45S_Trex_150	GGGCGCCGAGAGGCAAGGGGCGGGGACGGGCGGTGGCTCGCCTCGCGGCGGACCGCCCGC
45S_Trex_151	CCGCTCCCAAGATCCAACTACGAGCTTTTTAACTGCAGCAACTTTAATATACGCTATTGG
45S_Trex_152	AGCTGGAATTACCGCGGCTGCTGGCACCAGACTTGCCCTCCAATGGATCCTCGTTAAAGG
45S_Trex_153	ATTTAAAGTGGACTCATTCCAATTACAGGGCCTCGAAAGAGTCCTGTATTGTTATTTTTC
45S_Trex_154	GTCACTACCTCCCCGGGTCGGGAGTGGGTAATTTGCGCGCCTGCTGCCTTCCTTGGATGT
45S_Trex_155	GGTAGCCGTTTCTCAGGCTCCCTCTCCGGAATCGAACCCTGATTCCCCGTCACCCGTGGT
45S_Trex_156	CACCATGGTAGGCACGGCGACTACCATCGAAAGTTGATAGGGCAGACGTTCGAATGGGTC
45S_Trex_157	GTCGCCGCCACGGGGGGCGTGCGATCGGCCCGAGGTTATCTAGAGTCACCAAAGCCGCCG
45S_Trex_158	GCGCCCGCCCCCCGGCCGGGGCCGGAGAGGGGCTGACCGGGTTGGTTTTGATCTGATAAA
45S_Trex_159	TGCACGCATCCCCCCCGCGAAGGGGGTCAGCGCCCGTCGGCATGTATTAGCTCTAGAATT
45S_Trex_160	ACCACAGTTATCCAAGTAGGAGAGGAGCGAGCGACCAAAGGAACCATAACTGATTTAATG
45S_Trex_161	AGCCATTCGCAGTTTCACTGTACCGGCCGTGCGTACTCAGACATGCATGGCTTAATCTTT
45S_Trex_162	GAGACAAGCATATGCTACTGGCAGGATCAACCAGGTAGGTAAGGTAGAGCGCGGCGAGGC
45S_Trex_163	CCCGACGCGGCCGGACGGCCGGCCGGGGGGCCTCGCGAGGACGGGCCCGGCGCCCCGCAA
45S_Trex_164	GCGAGGAGGACGACGGACGGACGGACGGGCCGCGGACGGGCGGACGGGAGGGAGCGAGCG
45S_Trex_165	GGCGCGGGGGCGGCGGCCGGGACCGGTGGGGCCGGGGCGGGGCGCGGCGAACCGGACGCC
45S_Trex_166	CCAACCACCCGCCCCCCACGCGACACGACCACCGGGGCCCCGCGCCACAGACCCGCGACG
45S_Trex_167	CTTCTTCGTCGCGCCCGCCCGCGAGGAGGCGGACGGCCCGACCCGCGCCCGGCGGCCGGG
45S_Trex_168	AGGGACCGGCGGCCACGCGCGCGCGCGCGCGGCCGGCGCCCGCGGGCGGCGGCGAGGCGG
45S_Trex_169	GGACGGCGCTCCGCCCGCCCCGCGGGGCGGCCCCGACGTCCGGGCGGCGAGCGAGAGGCG
45S_Trex_170	GACCGCGGTGCCCGGCCCGGGGACAGTCGCGCCGTGCGGCCGCAGCGCCCGCGCACCGGT
45S_Trex_171	CCGGTCGAGGGCCCGGGGCCCGGCCGAAGCCCGGCTCCGAGCCCCGCCGGCGGGCGCGGG
45S_Trex_172	CGCAGGGGTGGCACACGCCACACGACGGCCAAGGGAGGGCCGACCGAGGCCGGCCGGCGC

45S_Trex_173	GCCCGCCCCCGCCCGGGACGGGGACCGCGACCGGGGCCGAGGCCCCGGCCCGGGCCCCA
45S_Trex_174	CCCCCCGACCCGGGGAAAGGGCGAGCGACCGGCAAGGCGGAGGTCGACCCACGCCACACG
45S_Trex_175	TCGCACGAACGCCTGTCCGGCAGGGACCACCGGGCCGCGCTCGGGCGCACGCGCGCGCCG
45S_Trex_176	AACGGGGCGACGCCACGCGGGGAGGACGGGCTCTCCCCGACGCCGACGCCCGGGACGGAC
45S_Trex_177	GCCTCGGGGAAGGGCCGCGGCAGGCCCGGGAAGCGAGGCGCACCCGGGGGACGCGCCGAC
45S_Trex_178	CCGGTTCGGAAGAGCGGGCCGGGAGAAGACGAGAGACCACGGGCGAGGCCGGGGCGACGG
45S_Trex_179	GGAAGGCGCGAGAAAGGCGGCCGGCGGGGAAGGGGACGCCACGGGGACCCCTCGAGCGCG
45S_Trex_180	GCCGACCGCGGCCGGGACACACGCGCGGGGCCTCACCGCCGCCGGCGGCACCGCGCGGCA
45S_Trex_181	CCCGGGGCGGCCGACCGGCCCTCGGCGATCCCCGCGGCTGCCCCCACCACCGCCGCCGCA
45S_Trex_182	GTCGCGGCCGGTCCCCCGGAACCGTCTCCTCCCCCGCACGCGCCGCAGGCCGACCCCCGG
45S_Trex_183	AACCCTCCGGGAAGCCCACCGGGCCCCACGCGGGGCGCCACCGACCCGGTCCCCAAGGCG
45S_Trex_184	CGCGCCGGGGGACGCGGACGCCGGGCCGATCAGTGGCCGGCGGCGGCGCCCCACGAGGCG
45S_Trex_185	GTGCCGGGTTCGGTCCCAGGCGGGGCCACCAACGGACGTGAAGCCGGTGAGCCGCTCGGG
45S_Trex_186	GGGAAGAAGAGGATCGGCGGGCGGCGGGCGGGGAAGAGGGCACAGACGGGCGAGGGCCGG
45S_Trex_187	GGACCGCGAGGGCAAGGGCACCCGGGAGCCCGCAGAGGCGGCGGCTCGGGGAGAAACCTC
45S_Trex_188	AGGCACGGCCGGGCCACCAGGAAAACACGGCCGCGGGATCCCACCGCCACAGACACGAGG
45S_Trex_189	GCGGTCCCGCGGCGCCCCGCCTGGGACGCCGGACGGCCCTCGGCCCCCACCGAGAACCGC
45S_Trex_190	CTCGCGAGCCCCGGGGCCCCGCCACCGGGGGCCCCGGAGCGACCGCAGCCACGAACCCGA
45S_Trex_191	CACGCCCGCACCACCGTCGCTCGTGATTCTCGTCCATCCTCCGACCCGGTCCCGCTCCGG
45S_Trex_192	GAGACCGGCGCGCCCCCACCGTGGGACGCTTTCCCAGGGCCAGGCGGGCCCGACCCCGTG
45S_Trex_193	CCACGCAAACGCGGTCGTCGGCACCGGTCACGACTCGGCACGGGAGCGGGCGGAGAGCCG
45S_Trex_194	ACTCGCGGCGGAGGGGGTCACGCGCCGGACAGAGCGCCGGGCGCGCACACCCACCGCCCG
45S_Trex_195	CCGGCCGCCGCGTCCCAACCCGCTGGGAACGCCGGGCCCGGCCCGGCGGGATCCTCCCCC
45S_Trex_196	GACTCGGAAGGGGGAGGCGCGGGCCACAGTAGGCGACGAGCCGCACTCGGCCACCACCGC
45S_Trex_197	GGTGGCCGGCGGAACCCTCGCTTCTCCCCCCCAACCCCGTCGAGGGGGAAGCGGAGGAGG
45S_Trex_198	GTCCTCTGCGAGCGGGTCGCTACGGCAGCGCTACCATAACGGAGGCAGAGACAGAGGCGG
45S_Trex_199	CGGCCCGGGGGATCCGGTACCCCCAAGGCACGCCTCTCAGATCGCTAGAGAAGGCTTTTC
45S_Trex_200	TCACCGAGGGTGGGTCACACTCCCCCCACCCGCCAGCCGCTCCTCCTCGGGCCCGCAGAG

45S_Trex_201	GCGCCGAGGGACGCCTGGGGAAGGGAGGGGGCCCTGCGGTACGAGGAAACACCTGCGCGC
45S_Trex_202	GGCCACCTCGAGCGTTCGCGTTCAGGGCGGGGGCCCGGCCGGTGCGCGCGTGCGCGCAAC
45S_Trex_203	CCCACCAGGCCCCCCCGTCCACCCACCTCCTTCCTTCCGAGGCAGAGCGCCTCCGAAGTC
45S_Trex_204	AACCCACACACGACCGGTCGGAGGCAGAACGGCAGCCCCTCGGCGGCCGGCCGGCGCACG
45S_Trex_205	CGTCACACCGGCCCGAACCCACCGCGATCGCTCACACGGCCCGCGCGCACCCGCCAGAGG
45S_Trex_206	GGAGCACGGGACGTGCGCTCACCGAGAGCAGGCGGGCGCCCTTCCCCGCGTGGGAGGGGC
45S_Trex_207	GCGTCTCGTCTCGTCTCACTCAAACCGCCTCGAACCCCACACCGACGAGCTCCCTCAGGA
45S_Trex_208	CCCACGCGCGGACACCGCGGCGGCGACCGGAGGAGGGGGCGCCGGGGGCGGGAACGACAC
45S_Trex_209	ACCACCGTTCGGCCTCGGGCACCTGAGGGACAACCCGGAGCGCTCCAGGAGCACCGCAAG
45S_Trex_210	GGCCCAGGCGGAGCCGACGCTCGCGCAAACCCCCCGAGAGGGCAGCACGACGGGCCGGCG
45S_Trex_211	GGACGGCACCCCCACCGCCGCGGAGGGGGGCCGCCCGCAAGTCGACAACCACTGGAGGCG
45S_Trex_212	ACAGCGAGGGCTGTCTGCCGCGTCAGAGGACCCCGCCGGCCCGCCCCGCGACGCAGAAGG
45S_Trex_213	CGGCGGGCGGGACGGCGAGGTCGGGCCGGGGTCCGCACCCCACGCCTTCCCACACGCACC
45S_Trex_214	GCCGGCGGGCGGGGAGAGGAGAGACGAGGGGACCCCCGCGGGGCGGAGCGAGAAGGACGG
45S_Trex_215	TCCCGTTCGCCACGAACGTCCGCCCCTCGCCCGTCGCGGCTCGGACCCGGCCCGGGAGAG
45S_Trex_216	CACGACGTCACCACATCGATCACGAAGAGCCCCCCGGGAGCGGAGGCCGGCCGGCCGGCC
45S_Trex_217	AGCGAGCCGATCGGCTCCGGCCAACCCCCCACTCCGGGGAAGGGGCGGCGGACAACCCCG
45S_Trex_218	CGGAGACGAGAACGCCTGACACGCACGGCACGGAGCCAGCGGGGTGGGGTTGTCGCGGCC
45S_Trex_219	GCCCCGGGCGCCCGCAGCGGAGAGCGCACGGGGGCACGGTGGCCCTCGCCGCCTTCCCCG
45S_Trex_220	CCGCCCCCGGGTGGGTCAGAGACCCGGACCCGGGCCGGCACCGGGAGTCGGGACGCTCGG
45S_Trex_221	ACGCGCGAGAGAACAGCAGGCCCGCGGGCCCCGGCAGGCGGCTCAAGCAGGAGCGCGGCC
45S_Trex_222	GGCTAGCCGGGTCACCGGTAGGCCAGAGCCCCGCGCGCATCCGGAGGCCCAACCTCTCCA
45S_Trex_223	GCGACAGGTCGCCAGAGGACAGCGTGTCAGC
18S	
18s_Trex_1	CCTTGTTACGACTTTTACTTCCTCTAGATAGTCAAGTTCGACCGTCTTCTCAGCGCTCCG
18s_Trex_2	CCAGGGCCGTGGGCCGACCCCGGCGGGGCCGATCCGAGGGCCTCACTAAACCATCCAATC
18s_Trex_3	GGTAGTAGCGACGGGCGGTGTGTACAAAGGGCAGGGACTTAATCAACGCAAGCTTATGAC
18s_Trex_4	CCGCACTTACTGGGAATTCCTCGTTCATGGGGAATAATTGCAATCCCCGATCCCCATCAC

18s_Trex_5	GAATGGGGTTCAACGGGTTACCCGCGCCTGCCGGCGTAGGGTAGGCACACGCTGAGCCAG
18s_Trex_6	TCAGTGTAGCGCGCGTGCAGCCCCGGACATCTAAGGGCATCACAGACCTGTTATTGCTCA
18s_Trex_7	ATCTCGGGTGGCTGAACGCCACTTGTCCCTCTAAGAAGTTGGGGGACGCCGACCGCTCGG
18s_Trex_8	GGGTCGCGTAACTAGTTAGCATGCCAGAGTCTCGTTCGTTATCGGAATTAACCAGACAAA
18s_Trex_9	TCGCTCCACCAACTAAGAACGGCCATGCACCACCACCCACGGAATCGAGAAAGAGCTATC
18s_Trex_10	AATCTGTCAATCCTGTCCGTGTCCGGGCCGGGTGAGGTTTCCCGTGTTGAGTCAAATTAA
18s_Trex_11	GCCGCAGGCTCCACTCCTGGTGGTGCCCTTCCGTCAATTCCTTTAAGTTTCAGCTTTGCA
18s_Trex_12	ACCATACTCCCCCCGGAACCCAAAGACTTTGGTTTCCCGGAAGCTGCCCGGCGGGTCATG
18s_Trex_13	GGAATAACGCCGCCGCATCGCCGGTCGGCATCGTTTATGGTCGGAACTACGACGGTATCT
18s_Trex_14	GATCGTCTTCGAACCTCCGACTTTCGTTCTTGATTAATGAAAACATTCTTGGCAAATGCT
18s_Trex_15	TTCGCTCTGGTCCGTCTTGCGCCGGTCCAAGAATTTCACCTCTAGCGGCGCAATACGAAT
18s_Trex_16	GCCCCCGGCCGTCCCTCTTAATCATGGCCTCAGTTCCGAAAACCAACAAAATAGAACCGC
18s_Trex_17	GGTCCTATTCCATTATTCCTAGCTGCGGTATCCAGGCGGCTCGGGCCTGCTTTGAACACT
18s_Trex_18	CTAATTTTTTCAAAGTAAACGCTTCGGGCCCCGCGGGACACTCAGCTAAGAGCATCGAGG
18s_Trex_19	GGGCGCCGAGAGGCAAGGGGCGGGGACGGGCGGTGGCTCGCCTCGCGGCGGACCGCCCGC
18s_Trex_20	CCGCTCCCAAGATCCAACTACGAGCTTTTTAACTGCAGCAACTTTAATATACGCTATTGG
18s_Trex_21	AGCTGGAATTACCGCGGCTGCTGGCACCAGACTTGCCCTCCAATGGATCCTCGTTAAAGG
18s_Trex_22	ATTTAAAGTGGACTCATTCCAATTACAGGGCCTCGAAAGAGTCCTGTATTGTTATTTTTC
18s_Trex_23	GTCACTACCTCCCCGGGTCGGGAGTGGGTAATTTGCGCGCCTGCTGCCTTCCTTGGATGT
18s_Trex_24	GGTAGCCGTTTCTCAGGCTCCCTCTCCGGAATCGAACCCTGATTCCCCGTCACCCGTGGT
18s_Trex_25	CACCATGGTAGGCACGGCGACTACCATCGAAAGTTGATAGGGCAGACGTTCGAATGGGTC
18s_Trex_26	GTCGCCGCCACGGGGGGCGTGCGATCGGCCCGAGGTTATCTAGAGTCACCAAAGCCGCCG
18s_Trex_27	GCGCCCGCCCCCCGGCCGGGGCCGGAGAGGGGCTGACCGGGTTGGTTTTGATCTGATAAA
18s_Trex_28	TGCACGCATCCCCCCCGCGAAGGGGGTCAGCGCCCGTCGGCATGTATTAGCTCTAGAATT
18s_Trex_29	ACCACAGTTATCCAAGTAGGAGAGGAGCGAGCGACCAAAGGAACCATAACTGATTTAATG
18s_Trex_30	AGCCATTCGCAGTTTCACTGTACCGGCCGTGCGTACTCAGACATGCATGGCTTAATCTTT
5.8S	
5.8S_Trex_1	CGCTCAGACAGGCGTAGCCCCGGGAGGAACCCGGGGCCGCAAGTGCGTTCGAAGTGTCGA

5.8S_Trex_2	TGATCAATGTGTCCTGCAATTCACATTAATTCTCGCAGCTAGCTGCGTTCTTCATCGACG
5.8S_Trex_3	CACGAGCCGAGTGATCCACCGCTAAGAGTCGTACGAGGTCGATTTGGCGAGGGCGCTCCC
28S	
28S_Trex_1	GGACAAACCCTTGTGTCGAGGGCTGACTTTCAATAGATCGCAGCGAGGGAGCTGCTCTGC
28S_Trex_2	TACGTACGAAACCCCGACCCAGAAGCAGGTCGTCTACGAATGGTTTAGCGCCAGGTTCCC
28S_Trex_3	CACGAACGTGCGGTGCGTGACGGGCGAGGGGGCGGCCGCCTTTCCAGCCGCGCCCCGTTT
28S_Trex_4	CCCAGGACGAAGGGCACTCCGCACCGGACCCCGGTCCCGGCGCGCGGCGGGGCACGCGCC
28S_Trex_5	CTCCCGCGCGCGCGGGGCGCGTGGAGGGGGGGGCGGCCCGCCGGCGGGGACAGGCGGGGG
28S_Trex_6	ACCGGCTATCCGAGGCCAACCGAGGCTCCGCGGCGCTGCCGTATCGTTCCGCCTGGGCGG
28S_Trex_7	GATTCTGACTTAGAGGCGTTCAGTCATAATCCCACAGATGGTAGCTTCGCCCCATTGGCT
28S_Trex_8	CCTCAGCCAAGCACATACACCAAATGTCTGAACCTGCGGTTCCTCTCGTACTGAGCAGGA
28S_Trex_9	TTACCATGGCAACAACACATCATCAGTAGGGTAAAACTAACCTGTCTCACGACGGTCTAA
28S_Trex_10	ACCCAGCTCACGTTCCCTATTAGTGGGTGAACAATCCAACGCTTGGTGAATTCTGCTTCA
28S_Trex_11	CAATGATAGGAAGAGCCGACATCGAAGGATCAAAAAGCGACGTCGCTATGAACGCTTGGC
28S_Trex_12	CGCCACAAGCCAGTTATCCCTGTGGTAACTTTTCTGACACCTCCTGCTTAAAACCCAAAA
28S_Trex_13	GGTCAGAAGGATCGTGAGGCCCCGCTTTCACGGTCTGTATTCGTACTGAAAATCAAGATC
28S_Trex_14	AAGCGAGCTTTTGCCCTTCTGCTCCACGGGAGGTTTCTGTCCTCCCTGAGCTCGCCTTAG
28S_Trex_15	GACACCTGCGTTACCGTTTGACAGGTGTACCGCCCCAGTCAAACTCCCCACCTGGCACTG
28S_Trex_16	TCCCCGGAGCGGGTCGCGCCCGGCCGGCGCGCGGCCGGGCGCTTGGCGCCAGAAGCGAGA
28S_Trex_17	GCCCCTCGGGGCTCGCCCCCCCGCCTCACCGGGTCAGTGAAAAAACGATCAGAGTAGTGG
28S_Trex_18	TATTTCACCGGCGGCCCGCAGGGCCGGCGGACCCCGCCCCGGGCCCCTCGCGGGGACACC
28S_Trex_19	GGGGGGGCGCCGGGGGCCTCCCACTTATTCTACACCTCTCATGTCTCTTCACCGTGCCAG
28S_Trex_20	ACTAGAGTCAAGCTCAACAGGGTCTTCTTTCCCCGCTGATTCCGCCAAGCCCGTTCCCTT
28S_Trex_21	GGCTGTGGTTTCGCTGGATAGTAGGTAGGGACAGTGGGAATCTCGTTCATCCATTCATGC
28S_Trex_22	GCGTCACTAATTAGATGACGAGGCATTTGGCTACCTTAAGAGAGTCATAGTTACTCCCGC
28S_Trex_23	CGTTTACCCGCGCTTCATTGAATTTCTTCACTTTGACATTCAGAGCACTGGGCAGAAATC
28S_Trex_24	ACATCGCGTCAACACCCGCCGCGGGCCTTCGCGATGCTTTGTTTTAATTAAACAGTCGGA
28S_Trex_25	TTCCCCTGGTCCGCACCAGTTCTAAGTCGGCTGCTAGGCGCCGGCCGAGGCGAGGCGCCG

28S_Trex_26	CGCGGAACCGCGGCCCCGGGGGCGGACCCGGCGGGGGGGACCGGCCCGCGGCCCCTCCGC
28S_Trex_27	CGCCCGCCGCCGCCGCCGCGCGCCGAGGAGGAGGGGGGAACGGGGGGCGGACGGGGCCGG
28S_Trex_28	GGGGGTAGGGCGGGGGGACGAACCGCCCCGCCCCGCCGCCCGCCGACCGCCGCCGCCCGA
28S_Trex_29	CCGCTCCCCGCCCCCAGCGGACGCGCGCGCGACGAGACGTGGGGTGGGGGGGGGGCGCG
28S_Trex_30	CCGGCGCCCGCCGGGCTCCCCGGGGGCGGCCGCGACGCCCGCCGCAGCTGGGGCGATCCA
28S_Trex_31	CGGGAAGGGCCCGGCTCGCGTCCAGAGTCGCCGCCGCCGCCGGCCCCCCGGGTGCCCGGG
28S_Trex_32	CCCCCCTCGCGGGGGACCGTGCCCCCGCCGCCGGGGCCCCGCGGCGGGCCGCCGCCGGCC
28S_Trex_33	CCTGCCGCCCCGACCCTTCTCCCCCCGCCGCCGCCCCCACGCGGCGCTCCCCCGGGGAGG
28S_Trex_34	GGGGAGGACGGGGAGCGGGGGAGAGAGAGAGAGAGGGCGCGGGGCGGGGAGGGAGCGA
28S_Trex_35	GCGGCGCGCGCGGGGTGGGGCGGGGGAGGGCCGCGAGGGGGGTGCCCCGGGCGTGGGGGG
28S_Trex_36	GGCGGCGGCGCCTCGTCCAGCCGCGGCGCGCGCCCAGCCCCGCTTCGCGCCCCAGCCCGA
28S_Trex_37	CCGACCCAGCCCTTAGAGCCAATCCTTATCCCGAAGTTACGGATCCGGCTTGCCGACTTC
28S_Trex_38	CCTTACCTACATTGTTCCAACATGCCAGAGGCTGTTCACCTTGGAGACCTGCTGCGGATA
28S_Trex_39	TGGGTACGGCCCGGCGCGAGATTTACACCCTCTCCCCCGGATTTTCAAGGGCCAGCGAGA
28S_Trex_40	GCTCACCGGACGCCGCCGGAACCGCGACGCTTTCCAAGGCACGGGCCCCTCTCTCGGGGC
28S_Trex_41	GAACCCATTCCAGGGCGCCCTGCCCTTCACAAAGAAAAGAGAACTCTCCCCGGGGCTCCC
28S_Trex_42	GCCGGCTTCTCCGGGATCGGTCGCGTTACCGCACTGGACGCCTCGCGGCGCCCATCTCCG
28S_Trex_43	CCACTCCGGATTCGGGGATCTGAACCCGACTCCCTTTCGATCGGCCGAGGGCAACGGAGG
28S_Trex_44	CCATCGCCCGTCCCTTCGGAACGGCGCTCGCCCATCTCTCAGGACCGACTGACCCATGTT
28S_Trex_45	CAACTGCTGTTCACATGGAACCCTTCTCCACTTCGGCCTTCAAAGTTCTCGTTTGAATAT
28S_Trex_46	TTGCTACTACCACCAAGATCTGCACCTGCGGCGGCTCCACCCGGGCCCGCGCCCTAGGCT
28S_Trex_47	TCAAGGCTCACCGCAGCGGCCCTCCTACTCGTCGCGGCGTAGCGTCCGCGGGGCTCCGGG
28S_Trex_48	GGCGGGGAGCGGGGCGTGGGCGGGAGGAGGGGAGGAGGCGTGGGGGGGGGGCGGGGGAA
28S_Trex_49	GGACCCCACACCCCCGCCGCCGCCGCCGCCGCCGCCCTCCGACGCACACCACACGCGCGC
28S_Trex_50	GCGCGCGCGCCGCCCCCGCCGCTCCCGTCCACTCTCGACTGCCGGCGACGGCCGGGTATG
28S_Trex_51	GGCCCGACGCTCCAGCGCCATCCATTTTCAGGGCTAGTTGATTCGGCAGGTGAGTTGTTA
28S_Trex_52	CACACTCCTTAGCGGATTCCGACTTCCATGGCCACCGTCCTGCTGTCTATATCAACCAAC
28S_Trex_53	ACCTTTTCTGGGGTCTGATGAGCGTCGGCATCGGGCGCCTTAACCCGGCGTTCGGTTCAT

28S_Trex_54	CCCGCAGCGCCAGTTCTGCTTACCAAAAGTGGCCCACTAGGCACTCGCATTCCACGCCCG
28S_Trex_55	GCTCCACGCCAGCGAGCCGGGCTTCTTACCCATTTAAAGTTTGAGAATAGGTTGAGATCG
28S_Trex_56	TTTCGGCCCCAAGACCTCTAATCATTCGCTTTACCGGATAAAACTGCGTGGCGGGGGTGC
28S_Trex_57	GTCGGGTCTGCGAGAGCGCCAGCTATCCTGAGGGAAACTTCGGAGGGAACCAGCTACTAG
28S_Trex_58	ATGGTTCGATTAGTCTTTCGCCCCTATACCCAGGTCGGACGACCGATTTGCACGTCAGGA
28S_Trex_59	CCGCTACGGACCTCCACCAGAGTTTCCTCTGGCTTCGCCCTGCCCAGGCATAGTTCACCA
28S_Trex_60	TCTTTCGGGTCCTAACACGTGCGCTCGTGCTCCACCTCCCCGGCGCGGCGGGCGAGACGG
28S_Trex_61	GCCGGTGGTGCGCCCTCGGCGGACTGGAGAGGCCTCGGGATCCCACCTCGGCCGGCGAGC
28S_Trex_62	GCGCCGGCCTTCACCTTCATTGCGCCACGGCGGCTTTCGTGCGAGCCCCCGACTCGCGCA
28S_Trex_63	CGTGTTAGACTCCTTGGTCCGTGTTTCAAGACGGGTCGGGTGGGTAGCCGACGTCGCCGC
28S_Trex_64	CGACCCCGTGCGCTCGCTCCGCCGTCCCCCTCTTCGGGGGACGCGCGCGTGGCCCCGAGA
28S_Trex_65	GAACCTCCCCCGGGCCCGACGGCGCGACCCGCCCGGGGCGCACTGGGGACAGTCCGCCCC
28S_Trex_66	GCCCCCCGACCCGCGCGCGGCACCCCCCCCGTCGCCGGGGCGGGGGCGCGGGGAGGAGGG
28S_Trex_67	GTGGGAGAGCGGTCGCGCCGTGGGAGGGGTGGCCCGGCCCCCCCACGAGGAGACGCCGGC
28S_Trex_68	GCGCCCCCGCGGGGGAGACCCCCCTCGCGGGGGATTCCCCGCGGGGGTGGGCGCCGGGAG
28S_Trex_69	GGGGGAGAGCGCGGCGACGGGTCTCGCTCCCTCGGCCCCGGGATTCGGCGAGTGCTGCTG
28S_Trex_70	CCGGGGGGGCTGTAACACTCGGGGGGGGTTTCGGTCCCGCCGCCGCCGCCGCCGCCGCCA
28S_Trex_71	CCGCCGCCGCCGCCGCCGCCCCGACCCGCGCGCCCTCCCGAGGGAGGACGCGGGGCCGGG
28S_Trex_72	GGGCGGAGACGGGGGAGGAGGAGGACGGACGGACGGACGGACGGGGCCCCCCGAGCCACC
28S_Trex_73	TTCCCCGCCGGGCCTTCCCAGCCGTCCCGGAGCCGGTCGCGGCGCACCGCCGCGGTGGAA
28S_Trex_74	ATGCGCCCGGCGGCGGCCGGTCGCCGGTCGGGGGACGGTCCCCCGCCGACCCCACCCCCG
28S_Trex_75	GCCCCGCCCGCCCACCCCCGCACCCGCCGGAGCCCGCCCCCTCCGGGGAGGAGGAGGAGG
28S_Trex_76	GGCGGCGGGGGAAGGGAGGGCGGGTGGAGGGGTCGGGAGGAACGGGGGGCGGGAAAGATC
28S_Trex_77	CGCCGGGCCGCCGACACGGCCGGACCCGCCGCCGGGTTGAATCCTCCGGGCGGACTGCGC
28S_Trex_78	GGACCCCACCCGTTTACCTCTTAACGGTTTCACGCCCTCTTGAACTCTCTCTTCAAAGTT
28S_Trex_79	CTTTTCAACTTTCCCTTACGGTACTTGTTGACTATCGGTCTCGTGCCGGTATTTAGCCTT
28S_Trex_80	AGATGGAGTTTACCACCCGCTTTGGGCTGCATTCCCAAGCAACCCGACTCCGGGAAGACC
28S_Trex_81	CGGGCCCGGCGCGCCGGGGGCCGCTACCGGCCTCACACCGTCCACGGGCTGGGCCTCGAT

28S_Trex_82	CAGAAGGACTTGGGCCCCCCACGAGCGGCGCCGGGGAGCGGGTCTTCCGTACGCCACATG
28S_Trex_83	TCCCGCGCCCCGCCGCGGGGCGGGGATTCGGCGCTGGGCTCTTCCCTGTTCACTCGCCGT
28S_Trex_84	TACTGAGGGAATCCTGGTTAGTTTCTTCTCCTCCGCTGACTAATATGCTTAAATTCAGCG
5'ETS	
5ETS_Trex_1	GAGACAAGCATATGCTACTGGCAGGATCAACCAGGTAGGTAAGGTAGAGCGCGGCGAGGC
5ETS_Trex_2	CCCGACGCGGCCGGACGGCCGGCCGGGGGGCCTCGCGAGGACGGGCCCGGCGCCCCGCAA
5ETS_Trex_3	GCGAGGAGGACGACGGACGGACGGACGGGCCGCGGACGGGCGGACGGGAGGGAGCGAGCG
5ETS_Trex_4	GGCGCGGGGGCGGCGGCCGGGACCGGTGGGGCCGGGGCGGGGCGCGGCGAACCGGACGCC
5ETS_Trex_5	CCAACCACCCGCCCCCCACGCGACACGACCACCGGGGCCCCGCGCCACAGACCCGCGACG
5ETS_Trex_6	CTTCTTCGTCGCGCCCGCCCGCGAGGAGGCGGACGGCCCGACCCGCGCCCGGCGGCCGGG
5ETS_Trex_7	AGGGACCGGCGGCCACGCGCGCGCGCGCGGCCGGCGCCCGCGGGCGGCGGCGAGGCGG
5ETS_Trex_8	GGACGGCGCTCCGCCCGCCCCGCGGGGCGGCCCCGACGTCCGGGCGGCGAGCGAGAGGCG
5ETS_Trex_9	GACCGCGGTGCCCGGCCCGGGGACAGTCGCGCCGTGCGGCCGCAGCGCCCGCGCACCGGT
5ETS_Trex_10	CCGGTCGAGGGCCCGGGGCCCGGCCGAAGCCCGGCTCCGAGCCCCGCCGGCGGGCGCGGG
5ETS_Trex_11	CGCAGGGGTGGCACACGCCACACGACGGCCAAGGGAGGGCCGACCGAGGCCGGCCGGCGC
5ETS_Trex_12	GCCCGCCCCCGCCCGGGACGGGGGACCGCGACCGGGGCCGAGGCCCCGGCCCGGGCCCCA
5ETS_Trex_13	CCCCCCGACCCGGGGAAAGGGCGAGCGACCGGCAAGGCGGAGGTCGACCCACGCCACACG
5ETS_Trex_14	TCGCACGAACGCCTGTCCGGCAGGGACCACCGGGCCGCGCTCGGGCGCACGCGCGCGCCG
5ETS_Trex_15	AACGGGGCGACGCCACGCGGGGAGGACGGGCTCTCCCCGACGCCGACGCCCGGGACGGAC
5ETS_Trex_16	GCCTCGGGGAAGGGCCGCGGCAGGCCCGGGAAGCGAGGCGCACCCGGGGGACGCGCCGAC
5ETS_Trex_17	CCGGTTCGGAAGAGCGGGCCGGGAGAAGACGAGAGACCACGGGCGAGGCCGGGGCGACGG
5ETS_Trex_18	GGAAGGCGCGAGAAAGGCGGCCGGCGGGGAAGGGGACGCCACGGGGACCCCTCGAGCGCG
5ETS_Trex_19	GCCGACCGCGGCCGGGACACACGCGCGGGGCCTCACCGCCGCCGGCGGCACCGCGCGGCA
5ETS_Trex_20	CCCGGGGCGGCCGACCGGCCCTCGGCGATCCCCGCGGCTGCCCCCACCACCGCCGCCGCA
5ETS_Trex_21	GTCGCGGCCGGTCCCCCGGAACCGTCTCCTCCCCCGCACGCGCCGCAGGCCGACCCCCGG
5ETS_Trex_22	AACCCTCCGGGAAGCCCACCGGGCCCCACGCGGGGCGCCACCGACCCGGTCCCCAAGGCG
5ETS_Trex_23	CGCGCCGGGGGACGCGGACGCCGGGCCGATCAGTGGCCGGCGGCGGCGCCCCACGAGGCG
5ETS_Trex_24	GTGCCGGGTTCGGTCCCAGGCGGGGCCACCAACGGACGTGAAGCCGGTGAGCCGCTCGGG

5ETS_Trex_25	GGGAAGAAGAGGATCGGCGGGCGGCGGGCGGGGAAGAGGGCACAGACGGGCGAGGGCCGG
5ETS_Trex_26	GGACCGCGAGGGCAAGGGCACCCGGGAGCCCGCAGAGGCGGCGGCTCGGGGAGAAACCTC
5ETS_Trex_27	AGGCACGGCCGGGCCACCAGGAAAACACGGCCGCGGGATCCCACCGCCACAGACACGAGG
5ETS_Trex_28	GCGGTCCCGCGGCGCCCCGCCTGGGACGCCGGACGGCCCTCGGCCCCCACCGAGAACCGC
5ETS_Trex_29	CTCGCGAGCCCCGGGGCCCCGCCACCGGGGGCCCCGGAGCGACCGCAGCCACGAACCCGA
5ETS_Trex_30	CACGCCCGCACCACCGTCGCTCGTGATTCTCGTCCATCCTCCGACCCGGTCCCGCTCCGG
5ETS_Trex_31	GAGACCGGCGCGCCCCCACCGTGGGACGCTTTCCCAGGGCCAGGCGGGCCCGACCCCGTG
5ETS_Trex_32	CCACGCAAACGCGGTCGTCGGCACCGGTCACGACTCGGCACGGGAGCGGGCGGAGAGCCG
5ETS_Trex_33	ACTCGCGGCGGAGGGGGTCACGCGCCGGACAGAGCGCCGGGCGCGCACACCCACCGCCCG
5ETS_Trex_34	CCGGCCGCCGCGTCCCAACCCGCTGGGAACGCCGGGCCCGGCCCGGCGGGATCCTCCCCC
5ETS_Trex_35	GACTCGGAAGGGGGAGGCGCGGGCCACAGTAGGCGACGAGCCGCACTCGGCCACCACCGC
5ETS_Trex_36	GGTGGCCGGCGGAACCCTCGCTTCTCCCCCCCAACCCCGTCGAGGGGGAAGCGGAGGAGG
5ETS_Trex_37	GTCCTCTGCGAGCGGGTCGCTACGGCAGCGCTACCATAACGGAGGCAGAGACAGAGGCGG
5ETS_Trex_38	CGGCCCGGGGGATCCGGTACCCCCAAGGCACGCCTCTCAGATCGCTAGAGAAGGCTTTTC
5ETS_Trex_39	TCACCGAGGGTGGGTCACACTCCCCCCACCCGCCAGCCGCTCCTCCTCGGGCCCGCAGAG
5ETS_Trex_40	GCGCCGAGGGACGCCTGGGGAAGGGAGGGGGCCCTGCGGTACGAGGAAACACCTGCGCGC
5ETS_Trex_41	GGCCACCTCGAGCGTTCGCGTTCAGGGCGGGGGCCCGGCCGGTGCGCGCGTGCGCGCAAC
5ETS_Trex_42	CCCACCAGGCCCCCCCGTCCACCCACCTCCTTCCTTCCGAGGCAGAGCGCCTCCGAAGTC
5ETS_Trex_43	AACCCACACACGACCGGTCGGAGGCAGAACGGCAGCCCCTCGGCGGCCGGCCGGCGCACG
5ETS_Trex_44	CGTCACACCGGCCCGAACCCACCGCGATCGCTCACACGGCCCGCGCGCACCCGCCAGAGG
5ETS_Trex_45	GGAGCACGGGACGTGCGCTCACCGAGAGCAGGCGGGCGCCCTTCCCCGCGTGGGAGGGGC
5ETS_Trex_46	GCGTCTCGTCTCGTCTCACTCAAACCGCCTCGAACCCCACACCGACGAGCTCCCTCAGGA
5ETS_Trex_47	CCCACGCGCGGACACCGCGGCGGCGACCGGAGGAGGGGGCGCCGGGGGCGGGAACGACAC
5ETS_Trex_48	ACCACCGTTCGGCCTCGGGCACCTGAGGGACAACCCGGAGCGCTCCAGGAGCACCGCAAG
5ETS_Trex_49	GGCCCAGGCGGAGCCGACGCTCGCGCAAACCCCCCGAGAGGGCAGCACGACGGGCCGGCG
5ETS_Trex_50	GGACGGCACCCCCACCGCCGCGGAGGGGGGCCGCCCGCAAGTCGACAACCACTGGAGGCG
5ETS_Trex_51	ACAGCGAGGGCTGTCTGCCGCGTCAGAGGACCCCGCCGGCCCGCCCCGCGACGCAGAAGG
5ETS_Trex_52	CGGCGGGCGGGACGGCGAGGTCGGGCCGGGGTCCGCACCCCACGCCTTCCCACACGCACC

5ETS_Trex_53	GCCGGCGGGCGGGGAGAGGAGAGACGAGGGGACCCCCGCGGGGCGGAGCGAGAAGGACGG
5ETS_Trex_54	TCCCGTTCGCCACGAACGTCCGCCCCTCGCCCGTCGCGGCTCGGACCCGGCCCGGGAGAG
5ETS_Trex_55	CACGACGTCACCACATCGATCACGAAGAGCCCCCCGGGAGCGGAGGCCGGCCGGCCGGCC
5ETS_Trex_56	AGCGAGCCGATCGGCTCCGGCCAACCCCCCACTCCGGGGAAGGGGCGGCGGACAACCCCG
5ETS_Trex_57	CGGAGACGAGAACGCCTGACACGCACGGCACGGAGCCAGCGGGGTGGGGTTGTCGCGGCC
5ETS_Trex_58	GCCCCGGGCGCCCGCAGCGGAGAGCGCACGGGGGCACGGTGGCCCTCGCCGCCTTCCCCG
5ETS_Trex_59	CCGCCCCCGGGTGGGTCAGAGACCCGGACCCGGGCCGGCACCGGGAGTCGGGACGCTCGG
5ETS_Trex_60	ACGCGCGAGAGAACAGCAGGCCCGCGGGCCCCGGCAGGCGGCTCAAGCAGGAGCGCGGCC
5ETS_Trex_61	GGCTAGCCGGGTCACCGGTAGGCCAGAGCCCCGCGCGCATCCGGAGGCCCAACCTCTCCA
5ETS_Trex_62	GCGACAGGTCGCCAGAGGACAGCGTGTCAGC
ITS1	
ITS1_Trex_1	GACGACGCACCGGGAGGAGGCCCTTCCTGGCGCGGCACGTCCCCCCCCCCCCGCCCAAG
ITS1_Trex_2	AGGAGAGGGGGTTGCCTCAGGCCGGCCAGACGAGACAGCAAACGGGACCGGACTCCGGAG
ITS1_Trex_3	AGGGGTCGGAAGGTTTCACACCACGGGGAGGCGCGCGCCGCCCACGCGGGGGCGAGCGCG
ITS1_Trex_4	GACACCACCCCACAGGCGCCCGGGGGTTCCCGCCCCCACGGCGCGGGGCGCACGCCACAC
ITS1_Trex_5	GCGCGGCAGGCGCGCGACGGCCGCCGGGTAAAGCCCCCACCCGACGGCCGCCGCGGCGGC
ITS1_Trex_6	GGCGGCGGCGCGGCCCCGGCCGGGGAGCGGAGTCCGCGGTGGAGGCGCGGGAGGGGCCGG
ITS1_Trex_7	GCCCCTCCCGACGGGACTCCCCCGCGGGCCCACCACCGCCCCCGACCCACGGGCGGACGG
ITS1_Trex_8	GCGATCCCCCCAAGGGGTCTTTAAACCTCCGCGCCGGAACGCGCTAGGTACCTGGACGGC
ITS1_Trex_9	GGGGGGGCGGACGAGGAGGCGGGGGAGGGGACCGGCGTCCGGCCCCCGACCCTCGAGACG
ITS1_Trex_10	CCCTAGCGGGAAGGCCGGGGAGAGCGAGCGGGGCCGTGCCCGGCGGCGCGGAGCGGCGCG
ITS1_Trex_11	GCGGAGGCGACGGGAATCCGGCCGGCCCCGAAGACGGGGAGCCGGCGCGGCGGGGCCGGA
ITS1_Trex_12	CGACGGGCCCCGGCGGGGAGGAGGGCACCGAGACCCCCCCAGACCCGCCGCGACGCCGCC
ITS1_Trex_13	GAGAACCGCCCCCGCGCCCGCCGACACCCACGTCGTCGGGGCCGCGGCCGGGGACCGCTC
ITS1_Trex_14	CCCGCCGCCCGCCGGCCCCACGACACGCGCACACCAACGACACGCCCTTCTTTCTCTTTC
ITS1_Trex_15	TCTCTCTCTCTCTCTCTCCCCCGTCTCCCTCCCGAGTTCTCCGGCTCTCGCGGCCGGCGG
ITS1_Trex_16	GGCCGGGCGGCGAACGAACGAGCGAGCGAACGAACGGGCACGCGGGCCCCGCCCGCGCAC
ITS1_Trex_17	GCGCCGCGTCGCGGTGGGGGGGTGGGTGTGCGGAGGGAAGCGCGCGGCGGCGGCGGCGCC

ITS2	
ITS2_Trex_1	CGGACGGAATCGCGCCGGCCCGACCGCCCGCCCGACGCTCCGTCGGGAGACGGGCCCGGC
ITS2_Trex_2	GAGGGGGAGAGGCGACGGGAGAGAGAGCGCGCGGCCGGCGGCACCCCCGCGCCGCCCCGC
ITS2_Trex_3	CGGAGCGGGACGACCGGAGGGAGGGGCACGGGCCGGGGGCGGGACGGGCGCCGCACGCCC
ITS2_Trex_4	CGACCCGTCTCCCCCGCGGAGGTCGGGGGGACGGGTCCGAGGACGCGGCGGCGGAGCCGC
ITS2_Trex_5	CCCGCCCCGACGCGGAAGCTCGGGACGGGGCCCCGGCGCGGCGCGGCGCGGCCGCGAGCC
ITS2_Trex_6	GGAGGCGGGCGCGCGACGGCGGACGACACCGCGGCGTCCCGCGGGTCGCCGCCGGGGACA
ITS2_Trex_7	CGCGAACCCCGGCGCCGCGGCCACGGGCGCGGCCGGGCGGGCCGCGGGGCGGGCTCCCGG
ITS2_Trex_8	CCCCGGCCGACGCGCCGCGAGGCGAGCCGGGCGGGCGGGCGCGCGTACGCGCGGGGAGGG
ITS2_Trex_9	CGAGGAGGACGGGCGGGGCCTCGGAGGAGGGGCGGCGGGGAGGAGGAGGGGCGCGGGAGC
ITS2_Trex_10	GGCGGTCGGCCGGACGCCGGGCCGCCACCGGGGGCGGGCGGCGAACCGCGGCGACCGGGA
ITS2_Trex_11	CGCGCTCCCCCGACCCTCTCTCCCCGCCGGCACCCTTCCCCTTCCGGACCCGCCTTCCTC
ITS2_Trex_12	CTCCCCCACCACCACACCGCACGCAACACGCCCCCACCGCCGACGACGCGCGACGACGAC
ITS2_Trex_13	GACGACGACGGGCACGGGACCTTCCACCCGGCCGGGGCCGACGAACCCCGAACCCCGAGC
ITS2_Trex_14	CGCGCGCGGCGCGAGGGAGCCCCCCGAGGGAGGAACCCGGACCGCAGGCGGCGGCCACGG
ITS2_Trex_15	GAACTCGGCCCGAGCCGGCTCTCTCTTTCCCTCTCCGTCTTCGCGGGCGGCGGCGGCGCC
ITS2_Trex_16	GCCCTCCCCGTCTCTCTCAGCCGGGCGCGCCCCCCTCTCCCCCCCGCCACCCGACGCGTG
ITS2_Trex_17	ACCACGCAGGGCCCGCGGGGGGAGGGGGAAGGGGCGGGCGCGGCGGCAAGAGGAGGGCGG
ITS2_Trex_18	ACGCCGCCGGGTCTGCGCTTAGGGGGACGGAGGGCCCCCGGCGGGCCCTGCGAGGGAACC
ITS2_Trex_19	CCCAGCCGCGCACCCCGAGGAGCCCGGAGGCACCCCCGGGGGCGATTGATCGGCAAGCGA
3'ETS	
3ETS_Trex_1	CGCGAGGGCCGGGACCCCGGGTGGCCGCCCCACCGGGGCCCGCGCGGCCAACCCCCGGGA
3ETS_Trex_2	CGGGGACCGGCGGGCCACGGGCCCGGCTCGGCGCGGCCGCCTCCGCGGCTCCCAAACCAC
3ETS_Trex_3	GCTCCCCGGACCCCGTCCCGGCCCGGAGCGGACGAGCCGCCCCGGCGGTGAACGGGGAGG
3ETS_Trex_4	AGGCGGGAACCGAAGAAGCGGGGCGCGCCGACCGGGGTCGCGCGCCCTCCCCCCCACCCC
3ETS_Trex_5	CACCACCACGCCCGCGGTCGGCGGGAGAGGCCGGGAGGGAGGAAGACGAACGGAAGGACG
3ETS_Trex_6	GACGGCGCCGGACGCGCACGCCCCGCCGGGCCCCCCGCACGCACGCGCGCGCGCGCGC

Supplementary Table 2: List of all RT-qPCR primers used in this study.

**RT-qPCR primers**	**Sequence**
18S forward	CTTAGAGGGACAAGTGGCG
18S reverse	ACGCTGAGCCAGTCAGTGTA
28S forward	GGGTGGTAAACTCCATCTAAGG
28S reverse	GCCCTCTTGAACTCTCTCTTC
3'ETS forward	CCCGCTTCTTCGGTTCC
3'ETS reverse	GGCTCCCAAACCACGCT
5'ETS forward	AGCCTTCTCTAGCGATCTGA
5'ETS reverse	TACCATAACGGAGGCAGAG
5.8S forward	GTGCGTCGATGAAGAACGC
5.8S reverse	AGTGCGTTCGAAGTGTCGAT
5.8S - adjacent forward	GCAACCCCCTCTCCTCTTG
5.8S - adjacent reverse	CTCCCGACGACGCACCG
GAPDH forward	CAACAGCCTCAAGATCATCAG
GAPDH reverse	ATGGACTGTGGTCATGAGTC
ITS1 forward	TTCGTTCGCTCGCTCGTT
ITS1 reverse	CAACGACACGCCCTTCTTTC
ITS2 forward	GGCTGAGAGAGACGGGGA
ITS2 reverse	AGCCGGCTCTCTCTTTCC
NORAD-5' forward	AGCGAAGTCCCGAACGACGA
NORAD-5' reverse	TGGGCATTTCCAACGGGCCAA
NORAD-ND4 TREX forward	GGAGAGGAGTTGGAAGGAATG
NORAD-ND4 TREX reverse	TGTCCGCTATATACACAGTAGG
NORAD-ND4 CLIP forward	TTGTAGAAAGGCTGTCTTCTGA
NORAD-ND4 CLIP reverse	AGGCACGTGTCACTTAGAGC
RPS18 forward	ATCCCTGAAAAGTTCCAGCA
RPS18 reverse	CCCTCTTGGTGAGGTCAATG
U1 forward	ATACTTACCTGGCAGGGGAG
U1 reverse	CAGGGGGAAAGCGCGAACGCA

Supplementary Table 3: List of all reagents and materials used in this study.

Reagent and dilutions	Supplier	Catalogue number
**TREX**		
Invitrogen TURBO DNase and 10X Reaction Buffer	Fisher Scientific UK Ltd	10646175
UltraPure 10% SDS	Life Technologies Ltd	15553-035
Thermostable RNase H and RNase H Reaction Buffer	New England Biolabs UK Ltd	M0523S
Ambion RNase H	Fisher Scientific UK Ltd	AM2293
RNasin Plus RNase Inhibitor	Promega Ltd	N2611
Invitrogen Nuclease-Free Water	Fisher Scientific UK Ltd	10526945
1M Tris pH 7.0	Thermo Fisher Scientific	AM9850G
0.5M EDTA pH 8.0	Thermo Fisher Scientific	AM9260G
1M MgCl2	Thermo Fisher Scientific	AM9530G
Isopropanol	Fisher Scientific UK Ltd	P/7507/PB17
Ethanol	Fisher Scientific UK Ltd	E/0650DF/17
Acetone	Merck	34850-2.5L
Chloroform	Honeywell	650471-1L
5M NaCl	Thermo Fisher Scientific	AM9760G
TRI REAGENT LS (TRIZOL LS)	Sigma-Aldrich	T3934-200ML
TRI REAGENT (TRIZOL)	Sigma-Aldrich	93289-25ML
Nuclease-Free Water	Fisher Scientific UK Ltd	10526945
**MS sample preparation**		
Urea	Sigma-Aldrich	U1250-5KG
Ammonium bicarbonate	Sigma-Aldrich	A6141-500G
Thermo Fisher Scientific Pierce MS Grade Trypsin Protease	Fisher Scientific UK Ltd	13474189
Iodoacetamide	Biosciences	786-228
DTT, molecular biology grade (Dithiothreitol, Cleland's reagent)	Thermo Fisher Scientific	R0861
Vivacon 500, 30,000 MWCO Hydrosart	Sartorius	VN01H22
Pierce High pH Reversed-Phase Peptide Fractionation Kit	Life Technologies	84868
Empore™ Octadecyl C18 47mm Extraction Disks 2215	Empore™	66883-U
TFA, LC-MS Grade	Thermo Fisher Scientific	85183
Acetonitrile HPLC Far UV/ Gradient Grade	J.T.Baker	9012
Acetic Acid Glacial	Scientific Laboratory Supplies	200-580-7
Trypsin	Merck	T6567-1MG
**Library preparation and sequencing**		
PCR Add-on Kit for Illumina	Lexogen	20

RiboCop rRNA Depletion HMR V2 kit for ribosomal RNA + CORALL Total RNA-seq V2 kit	Lexogen	183-184
UDI 12 nt set B1	Lexogen	UDI12B_0001-0096
**Crosslinking and Immunoprecipitation (CLIP)**		
UltraPure 10% SDS	Life Technologies Ltd	15553-035
5M NaCl	Thermo Fisher Scientific	AM9760G
UltraPure™ 1 M Tris-HCI Buffer, pH 7.5	Thermo Fisher Scientific	15567027
Sodium Deoxycholate	Thermo Fisher Scientific	89905
IGEPAL CA-630, for molecular biology	Scientific Laboratory Supplies	I8896-50ML
Mini Protease Inhibitor Cocktail	Roche	4693124001
Rabbit IgG non-specific control (1:200 w/w)	Cell Signaling	2729
Topoisomerase1 Antibody (1:200 w/w)	Novus	NBP1-30481
RBMX Antibody (1:200 w/w)	Cell Signaling	14794
PUM1 Antibody (1:200 w/w)	Proteintech	26256-1-AP
UBR7 Antibody (1:200 w/w)	Cambridge Biosciences	A304-130A
RNase I	Thermo Fisher Scientific	EN0602
TURBO DNase	Thermo Fisher Scientific	AM2238
Proteinase K	Thermo Fisher Scientific	25530-049
SUPERase-In RNase Inhibitor	Thermo Fisher Scientific	AM2694
Protein G Dynabeads	Thermo Fisher Scientific	10003D
**RT-qPCR**		
EDTA	Thermo Fisher Scientific	15575-038
Proteinase K	Thermo Fisher Scientific	25530049
Power SYBR Green RNA-To-Ct<br>1 Step kit	Applied Biosystems	4389986
SDS	Thermo Fisher Scientific	15553027
TRI REAGENT LS (TRIZOL LS)	Sigma-Aldrich	T3934-200ML
TRI REAGENT (TRIZOL)	Sigma-Aldrich	93289-25ML
Tris HCl pH 8.0	Thermo Fisher Scientific	AM9855G
QuantiTect primer assay - Human UBR7	Qiagen	QT00002765
QuantiTect primer assay - Human RPP25L	Qiagen	QT01666252
**Tissue culture and RNAi**		
HCT116 colon carcinoma cells	ATCC	CCL-247
McCoy's 5A (Modified) Medium, GlutaMAX™ Supplement	Thermo Fisher Scientific	36600088
Trypsin/EDTA solution	Fisher Scientific UK Ltd	R-001-100
Gibco Fetal Bovine Serum	Thermo Fisher Scientific	10437028

Lipofectamine RNAi MAX	Thermo Fisher Scientific	13778150
Opti-MEM™ I Reduced Serum Medium for transfection	Thermo Fisher Scientific	31985062
ON-TARGETplus Non-targeting Control Pool siRNAs	Horizon discovery	D-001810-10-05
ON-TARGETplus gene-specific Smart pool siRNAs (HUMAN)	Horizon discovery	Various depending on the gene target
**Western blotting**		
NuPAGE 10%, Bis-tris, midi gels	Thermo Fisher Scientific	WG1201BOX
Immobilon-P PVDF (0.45μm)	Millipore	IPVH00010
NuPAGE MOPS running buffer	Thermo Fisher Scientific	NP0001
NuPAGE MES running buffer	Thermo Fisher Scientific	NP0002
NuPAGE transfer buffer	Thermo Fisher Scientific	NP0006
Topoisomerase1 Antibody (1:2000 v/v)	Novus	NBP1-30481
RBMX Antibody (1:1000 v/v)	Cell Signaling	14794
PUM1 Antibody (1:1000 v/v)	Proteintech	26256-1-AP
UBR7 Antibody (1:2000 v/v)	Cambridge Biosciences	A304-130A
TrueBlot® Anti-Rabbit IgG HRP (1:1000 v/v)	Rockland	18-8816-31
Rabbit IgG HRP linked (1:5000 v/v)	GE Healthcare	NA934
NuPAGE LDS sample buffer	Thermo Fisher Scientific	84788
DTT (Dithiothreitol)	Thermo Fisher Scientific	R0861
**Nascent RNA imaging**		
Anti-Brdu (1:200 v/v)	Merck	B2531-100UL
Anti-Nucleolin (1:100 v/v)	Abcam	ab22758
Alexa Fluor 488-conjugated Donkey Anti-Mouse IgG (H+L) (1:200 v/v)	Jackson ImmunoResearch	715-545-150
Alexa Fluor 647-conjugated Donkey Anti-Rabbit IgG (H+L) (1:200 v/v)	Jackson ImmunoResearch	711-605-152
Formaldehyde solution	Merck	F8775-500ML
SUPERase-In RNase Inhibitor	Thermo Fisher Scientific	AM2694
Bovine Serum Albumin (BSA)	Fisher Scientific UK Ltd	A3059-10G
5-Fluorouridine	Fisher Scientific UK Ltd	15494529
Hoechst 33258 (1:2000 v/v)	Sigma	861405
iBiDi u-Slide 18 Well flat, ibiTreat, Tissue Culture Treated slide	Thistle Scientific	81826
**Other commercial kits and reagent sets**		
Pierce™ BCA Protein Assay Kit	Thermo Fisher Scientific	23225
MycoAlert™ PLUS Mycoplasma Detection Kit	Lonza	LT07-705
West Pico PLUS Chemiluminescent reagent	Life Technologies Ltd	34577
