# Supplementary material for: TREX reveals proteins that bind to specific RNA regions in living cells
Source: Nat Methods. 2024 Feb 19;21(3):423–34. doi: 10.1038/s41592-024-02181-1 (PMC10927567; doi:10.1038/s41592-024-02181-1)
Supplement: Supplementary file 2 — Reporting Summary [file 41592_2024_2181_MOESM2_ESM.pdf]

nature portfolio

Corresponding author(s): Faraz K. Mardakheh

Last updated by author(s): Dec 28, 2023

Reporting Summary

Nature Portfolio wishes to improve the reproducibility of the work that we publish. This form provides structure for consistency and transparency in reporting. For further information on Nature Portfolio policies, see our Editorial Policies and the Editorial Policy Checklist.

Statistics

For all statistical analyses, confirm that the following items are present in the figure legend, table legend, main text, or Methods section.

n/a	Confirmed	
☐	☒	The exact sample size (*n*) for each experimental group/condition, given as a discrete number and unit of measurement
☐	☒	A statement on whether measurements were taken from distinct samples or whether the same sample was measured repeatedly
☐	☒	The statistical test(s) used AND whether they are one- or two-sided<br>*Only common tests should be described solely by name; describe more complex techniques in the Methods section.*
☐	☒	A description of all covariates tested
☐	☒	A description of any assumptions or corrections, such as tests of normality and adjustment for multiple comparisons
☐	☒	A full description of the statistical parameters including central tendency (e.g. means) or other basic estimates (e.g. regression coefficient) AND variation (e.g. standard deviation) or associated estimates of uncertainty (e.g. confidence intervals)
☐	☒	For null hypothesis testing, the test statistic (e.g. *F*, *t*, *r*) with confidence intervals, effect sizes, degrees of freedom and *P* value noted<br>*Give P values as exact values whenever suitable.*
☒	☐	For Bayesian analysis, information on the choice of priors and Markov chain Monte Carlo settings
☒	☐	For hierarchical and complex designs, identification of the appropriate level for tests and full reporting of outcomes
☒	☐	Estimates of effect sizes (e.g. Cohen's *d*, Pearson's *r*), indicating how they were calculated

Our web collection on statistics for biologists contains articles on many of the points above.

Software and code

Policy information about availability of computer code

Data collection	Thermo XCalibur (version 4.5) SP1 for mass spectrometry data collection;<br>Zeiss ZEN blue (version 3.5) for microscopy imaging data collection.
Data analysis	Maxquant (version 1.6.6.3); Perseus (version 1.6.2.3); DescTools (version 0.99.52); fastqc tool (versions 0.11.9 & 0.12.1); UMItools (versions 1.1.1 & 1.1.4); Trim-galore (version 0.6.5); STAR aligner (versions 2.7.9a and 2.7.10b); RSEM software (version 1.3.1); Cutadapt (version 4.4) ; SAMtools (version 1.18); deepTools bamCompare (version 3.5.2); IGV-Web (version 1.13.9); GraphPad PRISM (version 9.5.1); ImageJ (version 1.53t); PyMOL (version 2.5.4)

For manuscripts utilizing custom algorithms or software that are central to the research but not yet described in published literature, software must be made available to editors and reviewers. We strongly encourage code deposition in a community repository (e.g. GitHub). See the Nature Portfolio guidelines for submitting code & software for further information.

Data

Policy information about availability of data

All manuscripts must include a data availability statement. This statement should provide the following information, where applicable:

- Accession codes, unique identifiers, or web links for publicly available datasets
- A description of any restrictions on data availability
- For clinical datasets or third party data, please ensure that the statement adheres to our policy

All mass spectrometry raw files and their associated MaxQuant output files were deposited on ProteomeXchange Consortium, via the PRIDE partner repository, under the accession numbers PXD044643, PXD045385, and PXD044659 (https://proteomecentral.proteomexchange.org/). All RNA-sequencing FASTQ raw files were deposited to the NCBI BioProject portal, under the Project accession number PRJNA994065 (https://www.ncbi.nlm.nih.gov/bioproject/PRJNA994065). The Genome Reference Consortium Human Build 38 patch release 13 (GRCh38.p13) database was acquired from (https://www.ncbi.nlm.nih.gov/datasets/genome/GCF_000001405.39/). The Uniprot human reference proteome (UP000005640) database was acquired from (https://www.uniprot.org/proteomes/UP000005640).

Human research participants

Policy information about studies involving human research participants and Sex and Gender in Research.

Reporting on sex and gender	Not relevant to this study
Population characteristics	Not relevant to this study
Recruitment	Not relevant to this study
Ethics oversight	No ethics approval required for this study

Note that full information on the approval of the study protocol must also be provided in the manuscript.

Field-specific reporting

Please select the one below that is the best fit for your research. If you are not sure, read the appropriate sections before making your selection.

☒ Life sciences ☐ Behavioural & social sciences ☐ Ecological, evolutionary & environmental sciences

For a reference copy of the document with all sections, see nature.com/documents/nr-reporting-summary-flat.pdf

Life sciences study design

All studies must disclose on these points even when the disclosure is negative.

Sample size	In this study, pre-determining the sample size was not feasible. However, drawing from standard protocols in protein-interaction experiments like IP-MS, which typically employ three biological replicates, we hypothesized that TREX experiments would be analogous. Consequently, to attain sufficient statistical power, at least three replicate experiments were deemed necessary. To safeguard against potential loss of statistical power due to a failed replicate, we always conducted a minimum of four biological replicates in our TREX experiments.
Data exclusions	Amongst all the replicate samples analysed, only one replicate of the NORAD TREX experiment, and one replicate of the ITS2 TREX experiment were excluded, as these clearly failed the sample prep and not much proteins was detected in the resulting data afterwards. Accordingly, PCA analysis clearly showed that the replicates in question had failed and behaved as an outlier (see Extended Data Fig. 2c). No other data was excluded.
Replication	The TREX experiments were conducted four to five times each, and statistical analysis encompassed all replicates collectively. Every replication attempt succeeded across all targets, except for one replicate in the NORAD TREX experiment (with three remaining successful) and one in the ITS2 TREX experiment (with four remaining successful). Both failures were attributed to sample loss during preparation, due to lack of protein IDs. These unsuccessful replicates were subsequently excluded from the analyses, as detailed in Extended Data Fig. 2c, and Supplementary Datasets 2 and 9.
Randomization	The TREX samples were always randomly allocated for receiving treatment (+RNase H) or no treatment (control) at the digestion step.
Blinding	Where possible, the Perseus data analysis step in TREX was carried out blindly by a different researcher (not knowing which sample group is RNase H treated and which is not). Blinding was not implemented in other experiments, as typically the same researcher executed all the stages of an experiment, including analysis.

Reporting for specific materials, systems and methods

We require information from authors about some types of materials, experimental systems and methods used in many studies. Here, indicate whether each material, system or method listed is relevant to your study. If you are not sure if a list item applies to your research, read the appropriate section before selecting a response.

Materials & experimental systems

n/a	Involved in the study
☐	☒ Antibodies
☐	☒ Eukaryotic cell lines
☒	☐ Palaeontology and archaeology
☒	☐ Animals and other organisms
☒	☐ Clinical data
☒	☐ Dual use research of concern

Methods

n/a	Involved in the study
☒	☐ ChIP-seq
☒	☐ Flow cytometry
☒	☐ MRI-based neuroimaging

Antibodies

Antibodies used

Please note that the details of all antibodies used in this study, including their dilution, are now provided in Supplementary Table 3, as well as being listed below:

Anti-Brdu (Merck - B2531-100UL)(1:200 - IF);
Anti-Nucleolin (Abcam - ab22758) (1:100 - IF);
Alexa Fluor 488-conjugated Donkey Anti-Mouse IgG (H+L) (Jackson ImmunoResearch - 715-545-150) (1:200 - IF);
Alexa Fluor 647-conjugated Donkey Anti-Rabbit IgG (H+L) (Jackson ImmunoResearch - 711-605-152) (1:200 - IF);
Rabbit IgG non-specific control (Cell Signaling - 2729) (1:200 - CLIP);
Topoisomerase1 Antibody (Novus - NBP1-30481) (1:200 - CLIP, 1:2000 -WB);
RBMX Antibody (Cell Signaling - 14794) (1:200 - CLIP, 1:1000 - WB);
PUM1 Antibody (Proteintech - 26256-1-AP) (1:200 - CLIP, 1:1000 - WB);
UBR7 Antibody (Cambridge Biosciences - A304-130A) (1:200 - CLIP, 1:2000 - WB);
TrueBlot® Anti-Rabbit IgG HRP (Rockland - 18-8816-31) (1:1000 - WB);
Rabbit IgG HRP (GE Healthcare - NA934) (1:5000 - WB);

Validation

Anti-Brdu was validated in house and the control experiment is included in the manuscript (CX-5461 treatment, Extended Data Fig. 6b and 6c); Anti-Nucleolin was validated in house in our previous publication (Azman et al., EMBO J, 2023 - Fig.4); Topoisomerase1, PUM1, RBMX, and UBR7 antibodies for CLIP were also validated in-house by western blotting, showing specific immunoprecipitation of their targets at the correct molecular weights (Extended Data Fig. 2f, 2i, and 4f). All the secondary antibodies in use for IF or WB are validated by the manufacturer.

Eukaryotic cell lines

Policy information about cell lines and Sex and Gender in Research

Cell line source(s)	HCT116 colon carcinoma cells (ATCC - catalogue number: CCL-247)
Authentication	STR profiling (latest: 21/07/2023)
Mycoplasma contamination	Cells were regulary tested for mycoplasma and were always free of contamination
Commonly misidentified lines (See ICLAC register)	None used
