# Supplementary figures and images for: TREX reveals proteins that bind to specific RNA regions in living cells
Source: Nat Methods. 2024 Feb 19;21(3):423–34. doi: 10.1038/s41592-024-02181-1 (PMC10927567; doi:10.1038/s41592-024-02181-1)

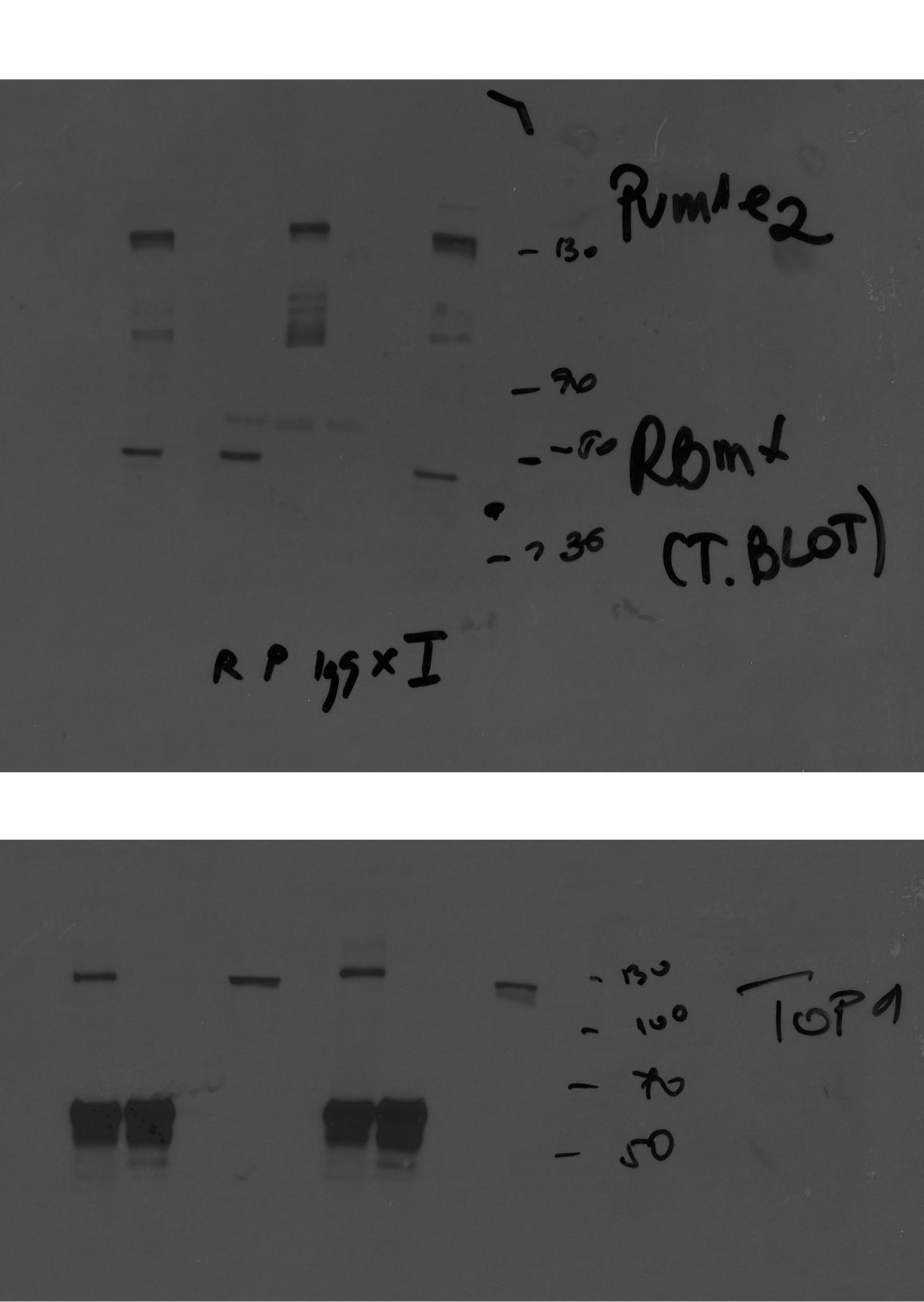

Supplement: Supplementary file 5 — Unprocessed western blots for Extended Data Fig. 2f,i. [file 41592_2024_2181_MOESM5_ESM.tif]

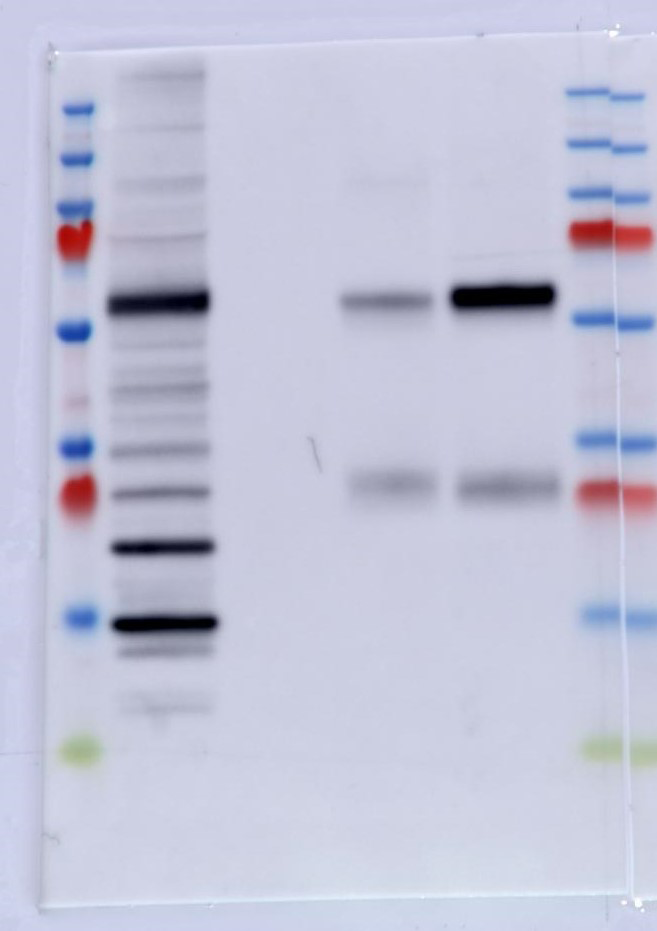

Supplement: Supplementary file 6 — Unprocessed western blots for Extended Data Fig. 4f. [file 41592_2024_2181_MOESM6_ESM.tif]
